# Microencapsulated *Bifidobacterium longum* subsp. *infantis* ATCC 15697 Favorably Modulates Gut Microbiota and Reduces Circulating Endotoxins in F344 Rats

**DOI:** 10.1155/2014/602832

**Published:** 2014-05-22

**Authors:** Laetitia Rodes, Shyamali Saha, Catherine Tomaro-Duchesneau, Satya Prakash

**Affiliations:** ^1^Biomedical Technology and Cell Therapy Research Laboratory, Department of Biomedical Engineering and Artificial Cells and Organs Research Centre, Faculty of Medicine, McGill University, 3775 University Street, Montreal, QC, Canada H3A 2B4; ^2^Faculty of Dentistry, McGill University, Montreal, QC, Canada H3A 2B2

## Abstract

The gut microbiota is a bacterial bioreactor whose composition is an asset for human health. However, circulating gut microbiota derived endotoxins cause metabolic endotoxemia, promoting metabolic and liver diseases. This study investigates the potential of orally delivered microencapsulated *Bifidobacterium infantis *ATCC 15697 to modulate the gut microbiota and reduce endotoxemia in F344 rats. The rats were gavaged daily with saline or microencapsulated *B. infantis* ATCC 15697. Following 38 days of supplementation, the treated rats showed a significant (*P *< 0.05) increase in fecal * Bifidobacteria* (4.34 ± 0.46 versus 2.45 ± 0.25% of total) and *B. infantis* (0.28 ± 0.21 versus 0.52 ± 0.12 % of total) and a significant (*P* < 0.05) decrease in fecal Enterobacteriaceae (0.80 ± 0.45 versus 2.83 ± 0.63% of total) compared to the saline control. In addition, supplementation with the probiotic formulation reduced fecal (10.52 ± 0.18 versus 11.29 ± 0.16 EU/mg; *P* = 0.01) and serum (0.33 ± 0.015 versus 0.30 ± 0.015 EU/mL; *P* = 0.25) endotoxins. Thus, microencapsulated *B. infantis* ATCC 15697 modulates the gut microbiota and reduces colonic and serum endotoxins. Future preclinical studies should investigate the potential of the novel probiotic formulation in metabolic and liver diseases.

## 1. Introduction

The human gut microbiota forms a large ecosystem consisting of approximately 10^14^ bacterial cells, a number 10 times greater than the number of human body cells [1]. The microbiome, which represents the collective genomes of the gut microbiota, is approximately 150 times larger than the human gene complement, with an estimated set of 3.3 million microbial genes [2]. The majority of the intestinal bacteria reside in the colon and belong to the Bacteroidetes, Firmicutes, and Actinobacteria phyla [2]. It is now well established that the gut microbiota is engaged in a dynamic interaction with the host, exerting essential protective, functional, and metabolic functions [3]. However, an imbalance in the composition of the gut microbiota, a state called gut dysbiosis, can disrupt the functions of the gut microbiota and impair human health [3].

Endotoxins are immunogenic molecules derived from the cell wall of Gram-negative bacteria that are produced in large quantities by the human gut microbiota [4]. Gut-derived endotoxins can enter the bloodstream, causing metabolic endotoxemia, a phenomenon characterized by low levels of circulating endotoxins [5–7]. Metabolic endotoxemia causes a mild and continuous induction of proinflammatory mediators, resulting in low-grade systemic inflammation [5–7]. This inflammatory state contributes to the progression of many human diseases, including obesity, type 2 diabetes, and liver, cardiovascular, and inflammatory bowel diseases [5–7]. Although the true incidence and prevalence of metabolic endotoxemia remain unknown, recent data suggests that metabolic endotoxemia occurs all over the globe, regardless of ethnicity [8]. Currently, there is no available intervention to reduce metabolic endotoxemia. Although many strategies have been developed to combat endotoxemia (e.g., antimicrobial therapies, endotoxins-binding proteins, and extracorporeal endotoxins absorbers), none is available for use in metabolic endotoxemia [9–11]. Thus, there is an urgent need for a novel intervention to reduce metabolic endotoxemia. Since the gut microbiota is the major source of endotoxins in metabolic endotoxemia, it may be a promising therapeutic target to reduce the condition.

Due to the inherent plasticity of the gut microbiota, probiotic biotherapeutics can promote human health by modulating the gut microbiota composition towards health-promoting bacterial populations [12]. Probiotics are “live microorganisms, which, when consumed in adequate amounts, confer a health benefit on the host” [12].* Bifidobacterium *spp. are common probiotic bacteria that are natural inhabitants of the human gastrointestinal tract and are present in many fermented dairy products [2, 12]. Sugar metabolism in* Bifidobacteria* produces high amounts of organic acids such as acetic and lactic acids [13]. In the colonic environment, acetic and lactic acids either can exert antimicrobial activities or be used in* de novo *fatty acid synthesis by other bacterial populations, providing multiple pathways that can modulate the gut microbiota composition [14–18]. Usually, the effect of probiotics formulations on the human gut microbiota composition is investigated primarily* in vitro* in human colonic models and* in vivo* in conventional or gnotobiotic rodents before any testing in humans [3]. Previous* in vitro* studies performed by our group have already demonstrated the potential of* Bifidobacterium longum* subsp.* infantis* (*B. infantis*) ATCC 15697 to modulate simulated human gut microbiota towards reduced colonic endotoxins concentrations [19]. The present study investigates the use of orally delivered alginate-poly-L-lysine-alginate (APA) microencapsulated* B. infantis* ATCC 15697 to modulate the gut microbiota composition and reduce endotoxemia in F344 conventional rats.

## 2. Materials and Methods

### 2.1. Animals, Experimental Design, and Treatment

Twelve F344 male rats were obtained from Charles River Laboratories (Wilmington, MA, USA) at five weeks of age (86–100 g). Rats were housed two per cage in a room with controlled temperature (22–24°C) and humidity. The rats were fed a standard diet and had free access to water throughout the trial. Following one-week acclimatization period, rats were randomly assigned, based on body mass values, into 2 groups (*n* = 6 per group): (1) control rats were administered 2 mL of 0.85% (w/v) NaCl and (2) treated rats were administered 2 mL of APA microencapsulated* B. infantis* ATCC 15697 at 5.5 × 10^9^ CFU/g dissolved in 0.85% (w/v) NaCl. Dosage was performed by intragastric gavage once a day. The treatment period lasted for 38 days. Animal mass was measured weekly. Fresh feces were collected weekly and stored at −80°C until analysis. Serum from rats that had been fasted for 16 h was collected biweekly by the lateral saphenous vein into Microtainer serum separator tubes from Becton Dickinson (Franklin Lakes, NJ, USA). Serum was obtained by allowing the blood to clot for a minimum of 30 min and centrifugation for 5 min at 10000 g. Serum samples were stored at −80°C until analysis. The rats were euthanized by CO_2_ asphyxiation and blood was withdrawn by cardiac puncture. Animal maintenance and experimental procedures complied with the Animal Care Committee of McGill University.

### 2.2. Bacterial Strain and Culture Conditions

*B. infantis* ATCC 15697 was purchased from Cedarlane Laboratories (Burlington, ON, Canada). The bacterial strain was stored at −80°C in de Man, Rogosa, and Sharpe (MRS, Fisher Scientific, Ottawa, Canada) broth containing 20% (v/v) glycerol. An MRS agar plate was streaked from the frozen stock and incubated at 37°C under anaerobic conditions for 24 h. One colony from the MRS agar plate was propagated into MRS broth and incubated at 37°C for 24 h. A 1% (v/v) inoculum was further passaged daily in MRS broth at 37°C. Bacterial cell viability was determined on MRS agar triplicate plates. Incubation was performed in anaerobic jars with anaerobe atmosphere-generating bags (Oxoid, Hampshire, United Kingdom) for 72 h at 37°C.

### 2.3. Microencapsulation Procedure

Microencapsulation of* B. infantis* ATCC 15697 was performed according to the standard protocol [20]. Briefly, the microcapsules were formed using an Inotech Encapsulator IER-20 (Inotech Biosystems International, Rockville, MD, USA) with a nozzle of 300 *μ*m in diameter under sterile conditions, as previously described [21]. Bacterial cells were released from the microcapsules by homogenizing capsules in 0.1 M sodium citrate.

### 2.4. Quantification of Fecal Bacterial Populations

Frozen feces were thawed and homogenized at a ratio of 0.1% (w/v) of feces in the ASL buffer provided with the QIAamp DNA stool Mini Kit (Qiagen, Toronto, ON, Canada). DNA was further extracted following the manufacturer's kit instructions and stored at −20°C. The quantification of bacterial populations was carried out by Real-Time- (RT-) PCR using the Eco Real-Time PCR System (Illumina Inc., San Diego, CA, USA) and the ROX RT-PCR Master Mix (2X) (Fisher Scientific), as previously described [21]. Enumeration of Enterobacteriaceae,* Escherichia coli*, Bacteroidetes,* Bacteroides *sp.*-Prevotella* sp., Actinobacteria,* Bifidobacterium *sp.,* B. infantis*, Firmicutes, and* Lactobacillus* sp. was performed using specific RT-PCR primer sequences (Table 1) [22–27]. RT-PCR signals specific to a bacterial group were normalized to the RT-PCR signals of total bacteria. The abundance of* Bifidobacteria* other than* B. infantis* was calculated as the difference between the abundance of total* Bifidobacteria* and that of* B. infantis*. A nontemplate control was included in each assay to confirm that the Ct value generated by the lowest DNA concentration was not an artifact. To determine the specificity of the DNA amplification reactions, a melt curve analysis was carried out after amplification.

### 2.5. Endotoxins Quantification

Fecal and serum endotoxin concentrations were measured using the ToxinSensor Chromogenic* Limulus* amebocyte lysate (LAL) Endotoxin Assay Kit from GenScript (Piscataway, NJ, USA) under sterile conditions. For colonic analysis, fecal samples were diluted at a ratio of 15% (w/v) in endotoxin-free water. The samples were then vortexed for 1 min and the homogenate was centrifuged at 10000 g for 10 min. The endotoxins-containing supernatant was further stored at −20°C until endotoxins quantification. For serum analysis, serum was diluted at 1 : 10 (v/v) in endotoxin-free water. Samples were assayed at different dilutions and plotted against a standard curve of endotoxins concentrations (0.0, 0.1, 0.25, 0.5, and 1.0 EU/mL), according to the manufacturer's instructions.

### 2.6. Quantification of Fecal Organic Acids

Fecal butyric, acetic, and lactic acids concentrations were determined by high-performance liquid chromatography (HPLC) using a Varian 335 model (Agilent, Fort Worth, TX, USA). Fecal samples were diluted at a ratio of 15% (w/v) in sterile distilled water. Then, the samples were vortexed for 1 min and the homogenate was centrifuged at 10000 g for 10 min. The organic acids-containing supernatant was stored at −20°C until HPLC analysis. The analysis was performed on a HPLC ion-exclusion column: Rezex ROA-Organic Acid H+ (8%), 25 × 0.46 cm, set up with SecurityGuard guard Cartridges (Phenomenex, Torrance, CA, USA). The HPLC system consisted of a ProStar 335 diode array detector set at 210 nm and a ProStar 410 autosampler monitored using the Varian Star 6 Chromatography Worstation (ProStar Version 6.0). Degassed 5 mM H_2_SO_4_ was used as the mobile phase at a flow rate of 0.2 mL/min. The injection volume was 10 *μ*L and the analysis was carried out at room temperature. Before analysis, samples were thawed, mixed at a ratio of 4 : 5 (v/v) with an internal standard of 50 mM 2-ethylbutyric acid, filtered through a 0.20 *μ*m PROgene nylon membrane (Ultident, St. Laurent, QC, Canada) directly into HPLC vials, and immediately sealed and analyzed. Calibration curves were generated using seven different concentrations of standards: 1, 5, 10, 25, 50, 75, and 100 mM for acetic acid (ACP, St Leonard, QC, Canada) and 0.6, 3, 6, 15, 30, 45, and 60 mM for lactic and butyric acids (Supelco, Bellefonte, PA, USA). The organic acids were identified by comparing each peak's retention time with those of standards.

### 2.7. Statistical Analysis

The experimental results are presented as the mean ± standard error of the mean (SEM) (*n* = 6). D'Agostino and Pearson normality test was performed to assess Gaussian distribution of the data. Bartlett's test was performed to assess homogeneity of variances. Statistical difference between the treatment groups (saline versus APA microencapsulated* B. infantis*) was analyzed at endpoint (day 38) using unpaired Student's* t*-test for parametric data or the Mann-Whitney test for nonparametric data. Correlations were performed using Pearson's correlation in the saline and APA microencapsulated* B. infantis* treatment groups at endpoint (day 38). Statistical significance was set at *P* < 0.05. All analyses were performed using the Prism software (Prism, Version 5.0, GraphPad Software, San Diego, CA, USA).

## 3. Results

### 3.1. Effect of APA Microencapsulated* B. infantis* ATCC 15697 on Fecal Bacterial Populations

**Figure 1:**
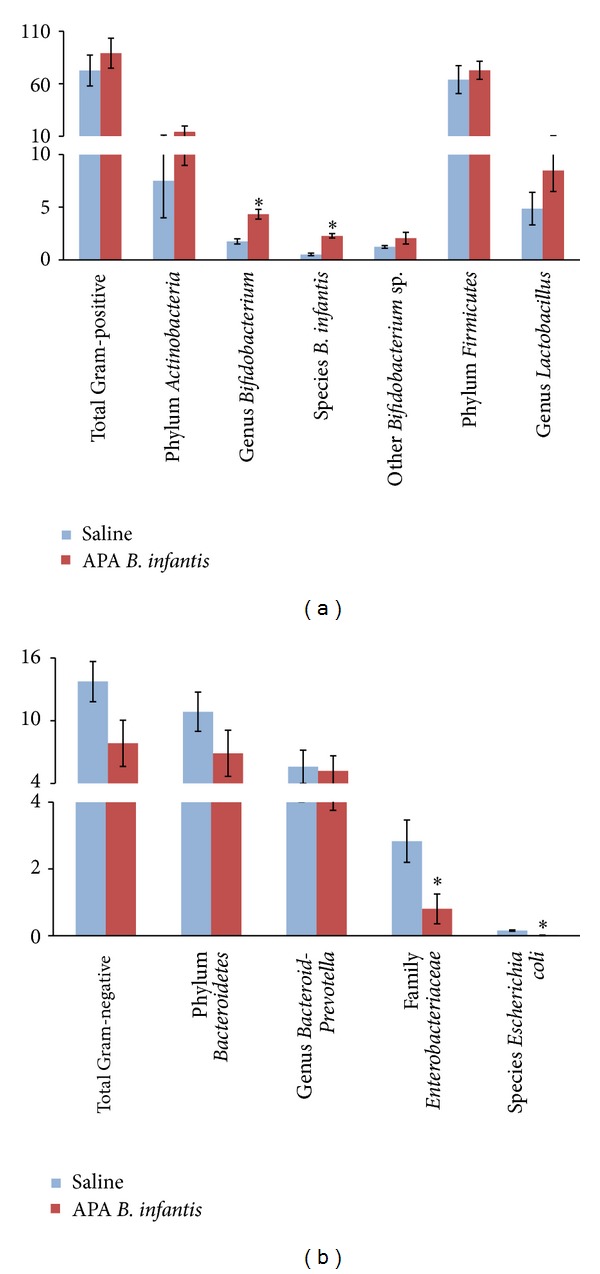
Effect of alginate-poly-L-lysine-alginate (APA) microencapsulated* B. infantis* ATCC 15697 supplementation on the abundance of fecal bacteria at endpoint (day 38): (a) bacteria that do not produce endotoxins and (b) potential endotoxins-producing bacteria. F344 rats were gavaged daily with APA microencapsulated* B. infantis* ATCC 15697 or saline during 38 days. Data represent the means ± SEM (*n* = 6) of the abundance of each bacterial group (mean percentage of total bacteria) at endpoint (day 38). Statistical analysis was performed using unpaired Student's* t*-test or the Mann-Whitney test. *Indicates statistical significance between treatment groups (*P* < 0.05).

The effect of orally administered APA microencapsulated* B. infantis *ATCC 15697 on fecal bacteria was investigated after 38 days of daily supplementation (Figure 1). Results showed that APA microencapsulated* B. infantis *ATCC 15697 significantly increased the abundance of bacterial populations that do not produce endotoxins. There was a significant increase in Gram-positive* Bifidobacteria *(4.34 ± 0.46 versus 2.45 ± 0.25% of total; *P* = 0.001) and* B. infantis *(0.28 ± 0.21 versus 0.52 ± 0.12% of total; *P* = 0.002), as compared to the saline control. In addition, oral administration of APA microencapsulated* B. infantis *ATCC 15697 significantly reduced the levels of potential endotoxins-producing bacteria including Gram-negative Enterobacteriaceae (0.80 ± 0.45 versus 2.83 ± 0.63% of total; *P* = 0.026) and* E. coli *(0.01 ± 0.01 versus 0.06 ± 0.02% of total; *P* = 0.026). Furthermore, there was a nonsignificant increase in the abundance of total Gram-positivebacteria (89.11 ± 14.27 versus 72.74 ± 14.69% of total; *P* = 0.497) and a nonsignificant decrease in total Gram-negative bacteria (7.84 ± 2.22 versus 13.75 ± 1.92% of total; *P* = 0.074) associated with APA microencapsulated* B. infantis *ATCC 15697 supplementation.

### 3.2. Effect of APA Microencapsulated* B. infantis* ATCC 15697 on the Concentration of Fecal Organic Acids

**Figure 2:**
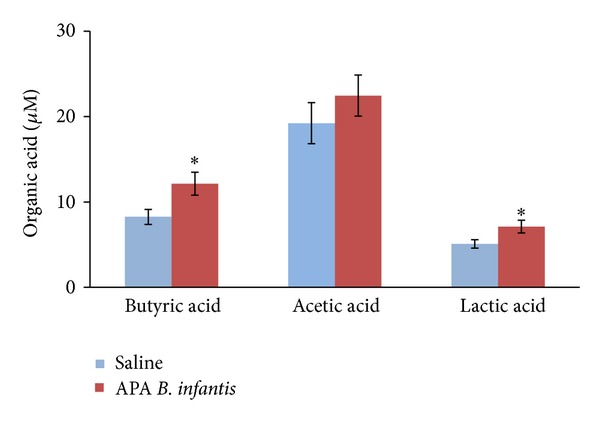
Effect of alginate-poly-L-lysine-alginate (APA) microencapsulated* B. infantis* ATCC 15697 supplementation on fecal organic acids concentrations at endpoint (day 38). F344 rats were gavaged daily with APA microencapsulated* B. infantis* ATCC 15697 or saline during 38 days. Data represent the means ± SEM (*n* = 6) of the concentration of organic acids per gram of wet feces at endpoint (day 38). Statistical analysis was performed using unpaired Student's* t*-test. *Indicates statistical significance between treatment groups (*P* < 0.05).

The effect of orally administered APA microencapsulated* B. infantis *ATCC 15697 on the levels of fecal organic acids was determined after 38 days of daily supplementation (Figure 2). Results showed that butyric (12.14 ± 1.34 versus 8.26 ± 1.34 *μ*M; *P* = 0.025) and lactic (7.11 ± 0.75 versus 5.09 ± 0.49 *μ*M; *P* = 0.025) acids were significantly increased following supplementation with APA microencapsulated* B. infantis *ATCC 15697, as compared to the saline control. The increase in acetic acid following supplementation with APA microencapsulated* B. infantis *ATCC 15697 was nonsignificant (22.46 ± 2.40 versus 19.23 ± 2.41 *μ*M; *P* = 0.365).

### 3.3. Effect of APA Microencapsulated* B. infantis* ATCC 15697 on Fecal and Serum Endotoxins Concentrations

**Figure 3:**
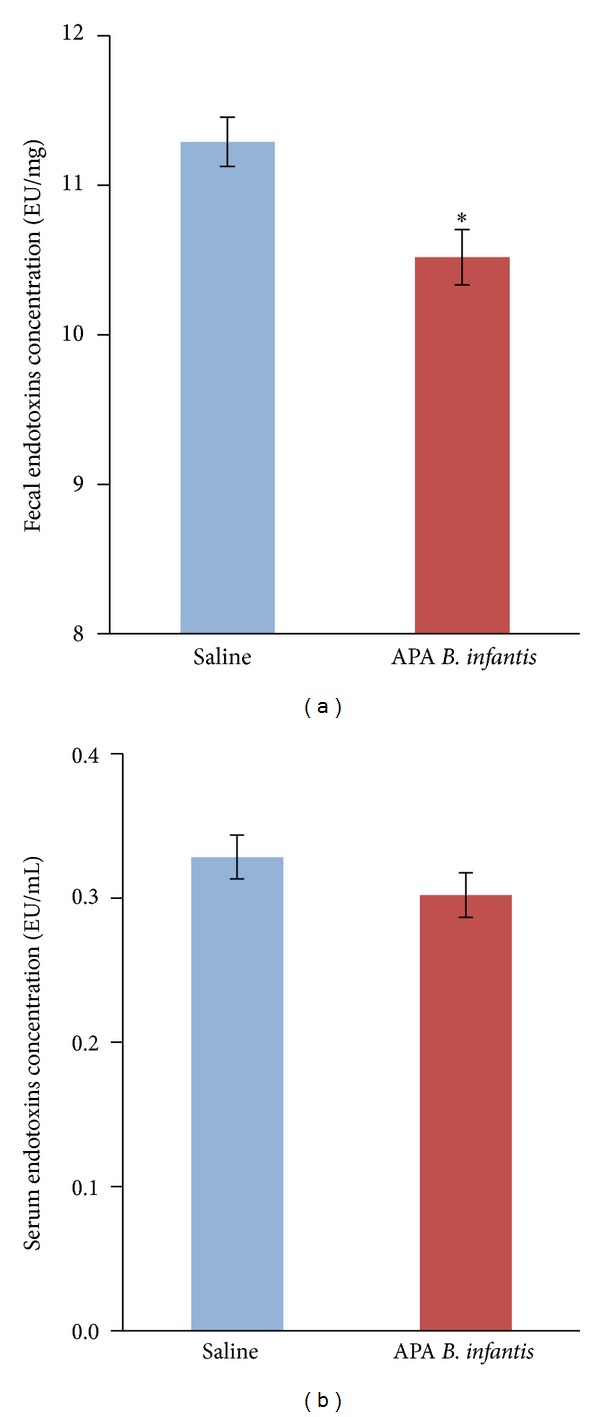
Effect of alginate-poly-L-lysine-alginate (APA) microencapsulated* B. infantis* ATCC 15697 on (a) fecal and (b) serum endotoxins concentrations. F344 rats were gavaged daily with APA microencapsulated* B. infantis* ATCC 15697 or saline during 38 days. Data represent the means ± SEM (*n* = 6) of the concentration of endotoxins at endpoint (day 38). Statistical analysis was performed using unpaired Student's* t*-test. *Indicates statistical significance between treatment groups (*P* < 0.05).

The effect of orally administered APA microencapsulated* B. infantis *ATCC 15697 on fecal and serum endotoxins was determined after 38 days of daily supplementation. Results showed that the probiotic formulation significantly reduced fecal endotoxins concentrations at endpoint (38 days) compared to the saline control, with a change averaging 7.34% (10.52 ± 0.18 versus 11.29 ± 0.16 EU/mg; *P* = 0.011; Figure 3(a)). Also, APA microencapsulated* B. infantis *ATCC 15697 supplementation decreased serum endotoxins concentrations with a change averaging 8.73%, but the effect was nonsignificant (0.33 ± 0.015 versus 0.30 ± 0.015 EU/mL; *P* = 0.252; Figure 3(b)).

### 3.4. Correlations between the Levels of Fecal Endotoxins and Bacterial Populations

**Figure 4:**
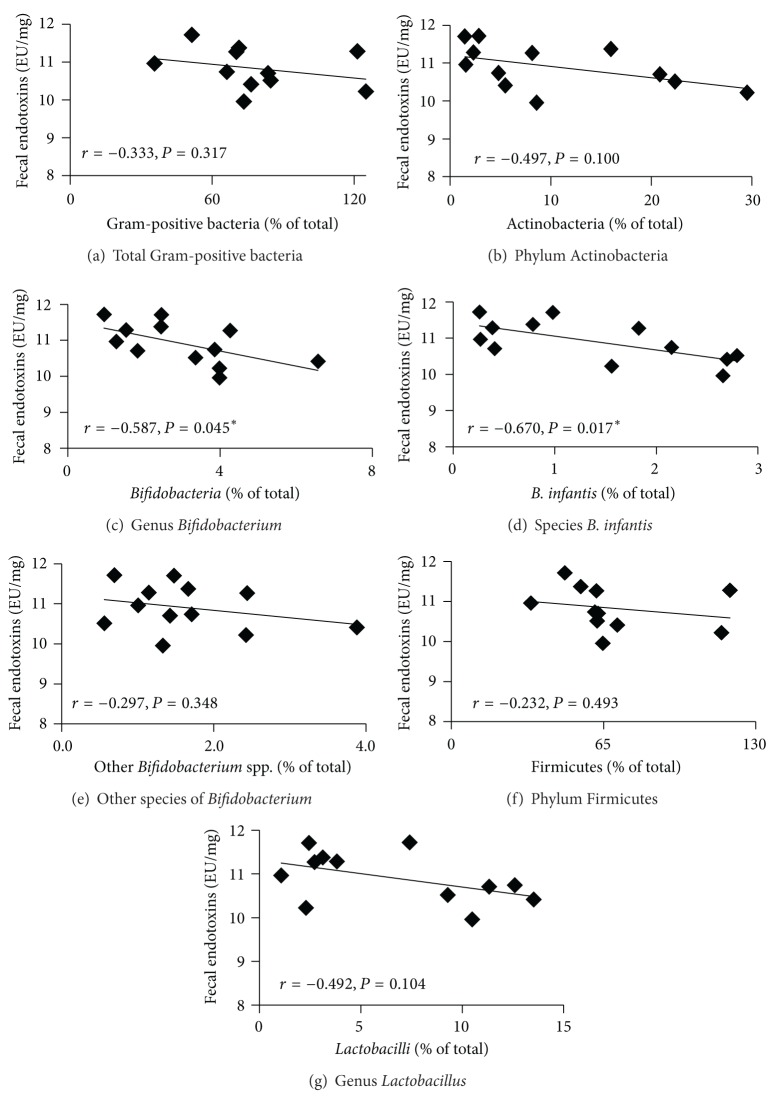
Correlations between the levels of fecal endotoxins and bacteria that do not produce endotoxins in F344 rats: (a) total Gram-positive bacteria, (b) phylum Actinobacteria, (c) genus* Bifidobacterium*, (d) species* B. infantis*, (e) other species of* Bifidobacterium*, (f) phylum Firmicutes, and (g) genus* Lactobacillus*. F344 rats were gavaged daily with APA microencapsulated* B. infantis* ATCC 15697 or saline during 38 days (*n* = 6). Correlations were performed at endpoint (day 38) using Pearson's correlation in the saline and APA microencapsulated* B. infantis* ATCC 15697 treatment groups. *Indicates statistical significance of the correlation (*P* < 0.05).

**Figure 5:**
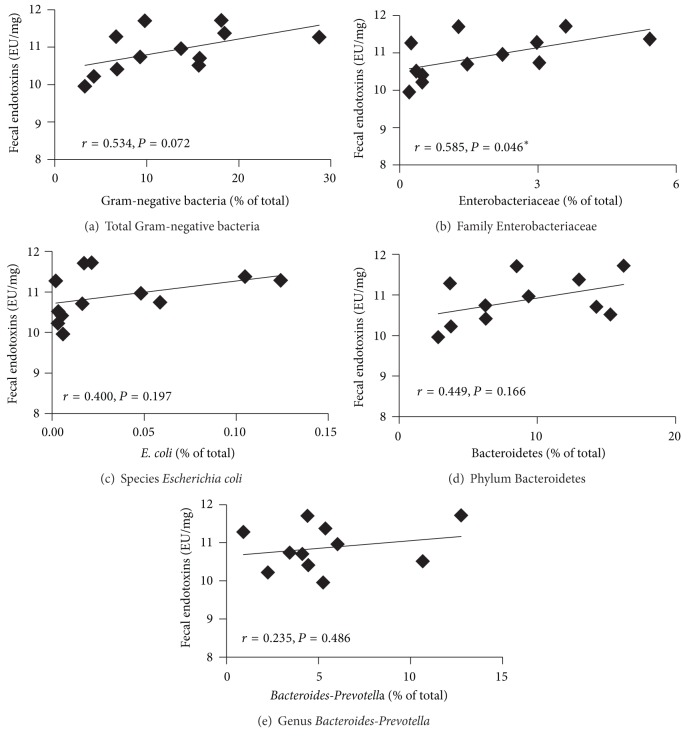
Correlations between the concentrations of fecal endotoxins and potential endotoxins-producing bacteria in F344 rats: (a) total Gram-negative bacteria, (b) family Enterobacteriaceae, (c) species* Escherichia coli*, (d) phylum Bacteroidetes, and (e) genus* Bacteroides-Prevotella*. F344 rats were gavaged daily with APA microencapsulated* B. infantis* ATCC 15697 or saline during 38 days (*n* = 6). Correlations were performed at endpoint (day 38) using Pearson's correlation in the saline and APA microencapsulated* B. infantis* ATCC 15697 treatment groups. *Indicates statistical significance of the correlation (*P* < 0.05).

To investigate the putative relationship between the levels of fecal endotoxins and bacteria that do not produce endotoxins (Figure 4) and potential endotoxins-producing bacteria (Figure 5), correlation analyses were performed. Results showed a significant negative correlation between fecal endotoxins concentrations and the abundance of Gram-positive* Bifidobacteria* (*r* = −0.587, *P* = 0.045) and* B. infantis* (*r* = −0.670, *P* = 0.017). Furthermore, there was a positive significant correlation between the levels of fecal endotoxins and Gram-negative Enterobacteriaceae (*r* = 0.585, *P* = 0.046).

### 3.5. Multicorrelation Analysis between the Levels of Fecal Organic Acids and Fecal/Serum Endotoxins and Fecal Bacterial Populations

**Figure 6:**
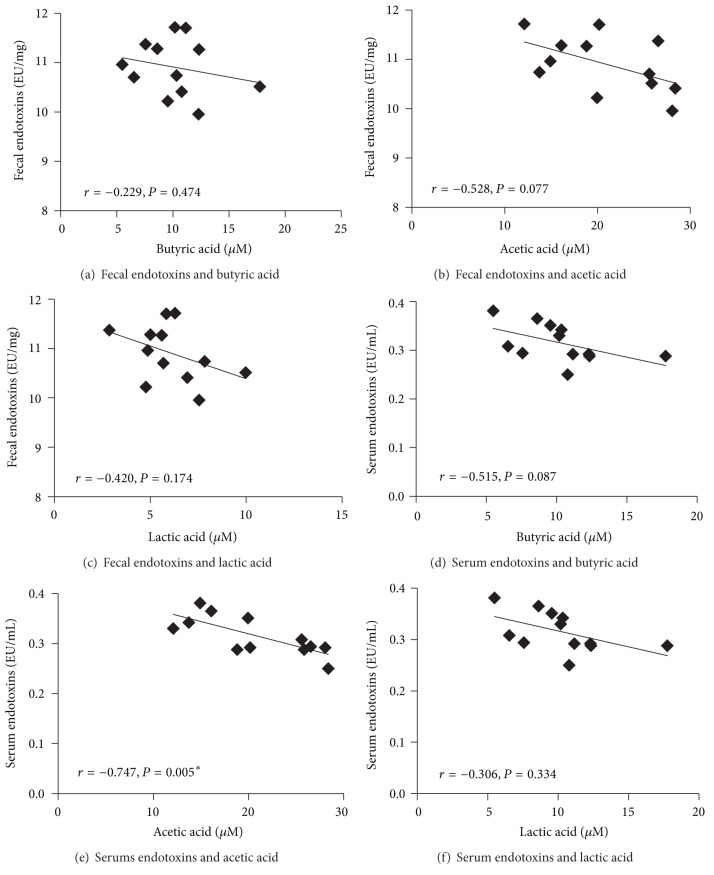
Correlations between the levels of fecal/serum endotoxins and fecal organic acids. F344 rats were gavaged daily with alginate-poly-L-lysine-alginate (APA) microencapsulated* B. infantis* ATCC 15697 or saline during 38 days (*n* = 6). Correlations were performed at endpoint (day 38) using Pearson's correlation in the saline and APA microencapsulated* B. infantis* ATCC 15697 treatment groups. *Indicates statistical significance of the correlation (*P* < 0.05).

Multicorrelation analysis was performed to investigate the putative relationship between the levels of fecal organic acids and fecal/serum endotoxins (Figure 6) and fecal bacterial populations (Table 2). Results showed that there was no significant correlation between the levels of fecal endotoxins and fecal butyric (*P* = 0.474), acetic (*P* = 0.077), and lactic (*P* = 0.174) acids. In addition, the level of serum endotoxins was significantly negatively correlated with fecal acetic acid concentration (*r* = −0.747; *P* = 0.005), while there was no significant correlation with fecal butyric (*P* = 0.087) and lactic (*P* = 0.334) acids concentrations. Furthermore, there was a significant negative correlation between the levels of fecal acetic acid and Enterobacteriaceae (*r* = −0.596; *P* = 0.041). There was also a significant positive correlation between the levels of fecal* B. infantis* and fecal butyric (*r* = 0.752; *P* = 0.005) and lactic (*r* = 0.696; *P* = 0.012) acids. Furthermore, the concentration of fecal lactic acid was significantly positively correlated with the abundance of* Lactobacilli *(*r* = 0.659; *P* = 0.020).

## 4. Discussion

It has been suggested to administer live probiotic bacterial cells in high doses in the colon to modulate the gut microbiota composition to promote human health [28]. As the cell viability of bacteria is hindered by the harsh conditions of the gastrointestinal tract (e.g., gastric acid and bile salts in the small intestine), microencapsulation has been extensively used to provide probiotic bacterial cells with a physical barrier to protect and deliver viable cells to the colon [21, 29]. Alginate microparticle systems have been used in particular because they are nontoxic, bioavailable, and cost-effective [21, 29]. Previous research has established the efficacy of APA microencapsulation as an effective delivery system to maintain the cell viability of* B. infantis* ATCC 15697 in the colon [21]. In addition,* in vitro* studies have demonstrated that* B. infantis* administration to the gut microbiota modulated gut bacterial populations towards reduced colonic endotoxins concentrations [19]. Endotoxins are potent immunomodulatory components derived from the cell wall of Gram-negative bacteria that can enter the blood circulation and cause metabolic endotoxemia [5–7]. The present study investigates the potential of orally delivered APA microencapsulated* B. infantis* ATCC 15697 to modulate the gut microbiota and lower endotoxemia in F344 rats.

The study shows that oral supplementation with APA microencapsulated* B. infantis* ATCC 15697 for 38 days significantly increases the levels of fecal* B. infantis* and* Bifidobacteria*. Although fecal bacteria do not exactly reproduce the gut microbiota composition [30], they represent a good indicator of the changes arising in the colon [3, 31]. In addition, APA microencapsulated* B. infantis *ATCC 15697 significantly reduced fecal Gram-negative Enterobacteriaceae and* E. coli* compared to the saline treatment, in agreement with previous studies [32]. Furthermore, supplementation with the probiotic bacterial formulation nonsignificantly reduced fecal Gram-negative bacteria and increased Gram-positive bacteria. The lack of statistical significance may be due to underestimated cell counts of Gram-negative and -positive bacteria, calculated based on the cell counts of Gram-negative Bacteroidetes and Enterobacteriaceae, and Gram-positive Firmicutes and Actinobacteria, respectively.

In addition, this study shows for the first time that supplementation with APA microencapsulated* B. infantis* ATCC 15697 significantly reduced fecal endotoxins concentrations* in vivo* compared to the saline treatment. Moreover, there was a significant negative correlation between fecal endotoxins concentrations and the abundance of* Bifidobacteria* and* B. infantis*, as observed by others [33, 34]. In addition, there was a significant positive correlation between the fecal levels of endotoxins and Gram-negative Enterobacteriaceae, suggesting that the decrease in Enterobacteriaceae might account for the endotoxins reduction, consistent with the findings of others [35, 36]. Furthermore, APA microencapsulated* B. infantis* ATCC 15697 nonsignificantly reduced serum endotoxins compared to the saline treatment, with a change averaging 8.73%. Endotoxins concentrations were determined using the chromogenic LAL assay, the most preferred method to quantify endotoxins in biological fluids. Although LAL assay can lead to erroneous endotoxins values due to variations in LAL preparations, cross-reactions, and low detection limits [37], our data is consistent with previous publications [32, 38]. The low number of animals included in our study (*n* = 6) may explain the nonsignificant statistical decrease in circulating endotoxins. Importantly, this serum endotoxins reduction may be of great importance physiologically, as a 10% change in serum endotoxins concentrations has been shown to induce significant consequences on systemic inflammation and human health during metabolic endotoxemia [39, 40]. It is also important to point out that the present study was performed in a healthy animal model, providing the proof of concept in a rat model. F344 rats are conventional and inexpensive rats that present a low level of circulating endotoxins. Future preclinical studies should confirm the potential of the probiotic bacterial formulation to lower circulating endotoxins in an animal model with metabolic endotoxemia, as observed in high-fat, diet-induced, obesity/type 2 diabetes/steatosis, ob/ob mice, and fatty Zucker (diabetic) rats.

It is well documented that probiotic bacteria such as* B. infantis* produce organic acids that can affect the gut microbiota composition [41, 42]. This study showed that APA microencapsulated* B. infantis* ATCC 15697 significantly increased fecal lactic, butyric, and acetic acids concentrations, as observed by others [43, 44]. In addition, there was a positive correlation between the fecal levels of* B. infantis* and organic acids, while the correlation between acetic acid and Enterobacteriaceae and serum endotoxins was negative. To date, there is no published data on the effect of acetic acid on the viability of Enterobacteriaceae, neither on gut endotoxins release and translocation. Nevertheless, previous studies have reported a negative relationship between the gut levels of Enterobacteriaceae and acetic acid [45, 46]. Altogether, this study suggests that oral supplementation with APA microencapsulated* B. infantis* ATCC 15697 increases the production of colonic organic acids, impeding the growth of endotoxins-producing bacteria such as Enterobacteriaceae. Future studies should investigate other mechanisms, including the production of exopolysaccharides and bacteriocins [47, 48].

## 5. Conclusions

This study demonstrates that supplementation with APA microencapsulated* B. infantis* ATCC 15697 reduces the levels of plasma endotoxins through a change in the gut microbiota characterized by reduced levels of endotoxins and Gram-negative Enterobacteriaceae and increased concentrations of Gram-positive* Bifidobacteria*. These changes may be mediated partly by the increased production of lactic, butyric, and acetic acids induced by the colonic delivery of* B. infantis* ATCC 15697. Thus, APA microencapsulated* B. infantis* ATCC 15697 is a promising probiotic bacterium to modulate favorably the gut microbiota and lower endotoxemia for use in metabolic endotoxemia. Further studies should confirm the present findings using an animal model with metabolic endotoxemia and underline the potential mechanism(s) of action.

## Figures and Tables

**Table 1:** Primers used for the quantification of fecal bacterial populations.

Target phylum or group	Primer	Sequence (5′ to 3′)	Reference
All bacteria	Bact-1369-F	CGGTGAATACGTTCCCGG	[22]
	Bact-1492-R	TACGGCTACCTTGTTACGACTT	

Phylum Firmicutes	Firm-928-F	TGAAACTCAAAGGAATTGACG	[23]
	Firm-1040-R	ACCATGCACCACCTGTC	

Genus* Lactobacillus *	Lact-05-F	AGCAGTAGGGAATCTTCCA	[24]
	Lact-04-R	CGCCACTGGTGTTCYTCCATATA	

Phylum Actinobacteria	Act-920-F3	TACGGCCGCAAGGCTA	[25]
	Act-1200-R	TRCTCCCCACCTTCCTCCG	

Genus *Bifidobacterium *	Bif-164-F	GGGTGGTAATGCCGGATG	[26]
	Bif-662-R	CCACCGTTACACCGGGAA	

Species *Bifidobacterium infantis *	F_inf_IS	CGCGAGCAAAACAATGGT T	[27]
	R_inf_IS	AACGATCGAAACGAACAATAGAGTT	

Phylum Bacteroidetes	CBF-798-F	CRAACAGGATTAGATACCCT	[23]
	CBF-967-R	GGTAAGGTTCCTCGCGTAT	

Genus *Bacteroides-Prevotella *	Bacter-11-F	CCTACGATGGATAGGGGTT	[22]
	Bacter-08-R	CACGCTACTTGGCTGGTTCAG	

Family Enterobacteriaceae	Eco1457-F	CATTGACGTTACCCGCAGAAGAAGC	[24]
	Eco1652-R	CTCTACGAGACTCAAGCT TGC	

Species *Escherichia coli *	E. coli-F	CATGCCGCGTGTATGAAGAA	[22]
	E. coli-R	CGGGTAACGTCAATGAGCAAA	

**Table 2:** Correlations between the levels of fecal organic acids and bacterial populations.

Bacteria (% of total)	Acetic acid (*μ*M)	Butyric acid (*μ*M)	Lactic acid (*μ*M)
Gram-positive	*r* = 0.193; *P* = 0.570	*r* = 0.136; *P* = 0.690	*r* = −0.084; *P* = 0.631
Phylum Actinobacteria	*r* = 0.458; *P* = 0.134	*r* = 0.176; *P* = 0.585	*r* = 0.019; *P* = 0.952
Genus *Bifidobacterium *	*r* = 0.511; *P* = 0.089	*r* = 0.436; *P* = 0.156	*r* = 0.345; *P* = 0.273
Species* B. infantis *	*r* = 0.509; *P* = 0.091	***r*** = **0.752**; ***P*** = 0.005*	***r*** = **0.696**; ***P*** = 0.012*
Other *Bifidobacterium *sp.	*r* = 0.341; *P* = 0.278	*r* = −0.058; *P* = 0.857	*r* = −0.157; *P* = 0.625
Phylum Firmicutes	*r* = 0.012; *P* = 0.973	*r* = 0.046; *P* = 0.892	*r* = −0.143; *P* = 0.675
Genus *Lactobacillus *	*r* = 0.363; *P* = 0.246	*r* = 0.286; *P* = 0.368	***r*** = **0.659**; ***P*** = 0.020*
Gram-negative	*r* = −0.157; *P* = 0.627	*r* = 0.054; *P* = 0.867	*r* = −0.155; *P* = 0.631
Phylum Bacteroidetes	*r* = −0.005; *P* = 0.989	*r* = 0.076; *P* = 0.825	*r* = 0.074; *P* = 0.829
Genus *Bacteroides-Prevotella *	*r* = −0.083; *P* = 0.809	*r* = 0.427; *P* = 0.190	*r* = 0.404; *P* = 0.218
Family Enterobacteriaceae	***r*** = **−0.596**; *P* = 0.041*	*r* = − 0.259; *P* = 0.416	*r* = −0.148; *P* = 0.647
Species *Escherichia coli *	*r* = −0.269; *P* = 0.389	*r* = −0.474; *P* = 0.119	*r* = −0.501; *P* = 0.097

F344 rats were gavaged daily with alginate-poly-L-lysine-alginate (APA) microencapsulated *B. infantis* ATCC 15697 or saline during 38 days (*n* = 6). Correlations were performed at endpoint (day 38) using Pearson's correlation in the saline and APA microencapsulated *B. infantis* ATCC 15697 treatment groups. *Indicates statistical significance of the correlation (*P* < 0.05).
